# Chemical Modification of Cellulose Microfibres to Reinforce Poly(methyl methacrylate) Used for Dental Application

**DOI:** 10.3390/ma13173807

**Published:** 2020-08-28

**Authors:** Joanna Taczała, Jacek Sawicki, Joanna Pietrasik

**Affiliations:** 1 Institute of Materials Science and Engineering, Lodz University of Technology, Stefanowskiego 1/15, 90-924 Lodz, Poland; joanna.taczala@dokt.p.lodz.pl; 2 Institute of Polymer and Dye Technology, Lodz University of Technology, Stefanowskiego 12/16, 90-924 Lodz, Poland

**Keywords:** cellulose microfibres, poly(methyl methacrylate), silanes, prosthetic dentistry

## Abstract

The mechanical properties of dental acrylic resins have to be improved in the case of a thin denture plate. This can be achieved by cellulose addition, playing the role of active filler. But to provide the excellent dispersion of cellulose microfibres within the hydrophobic polymer matrix, its surface has to be modified. Cellulose microfibres with average length from 8 to 30 μm were modified with octyltriethoxysilane and (3-methacryloxypropyl)methyldimethoxysilane. The latter also participated in the polymerisation reaction of methyl methacrylate. Dental composites were prepared following the general procedure provided by the supplier. The successful modification of the microfibres led to the improved compatibility of the cellulose and poly(methyl methacrylate). The fibres after modification were uniformly distributed within the matrix, resulting in the improved mechanical performance of obtained materials. Cellulose microfibres are good candidates for the dental materials to be used as the active filler. The simple and straightforward approach for the cellulose modifications with silanes provides good potential for its future practical application.

## 1. Introduction

Removable dentures are made of methacrylic polymers modified with various additives. The main ingredient of acrylic resins used in dental techniques is poly(methyl methacrylate), PMMA. Prostheses made of acrylic resin are durable enough to fall and bite forces. However, the problem occurs when the denture plate thickness has to be reduced.

Such a case occurs when the patient requires partial prosthetic reconstruction using a denture metal framework in which a single incisal tooth appears. The metal framework reduces the amount of space for the acrylic denture base plate and this leads to the tooth from the prosthesis being quickly broken [1]. This kind of denture should be replaced after around four years. However, patients report the need to repair after only a few months. Therefore, modification of the selected properties of the acrylic resin is necessary. 

Many additives were used to improve the strength of the prosthetic acrylic resin, such as varied oxides TiO_2_ [2,3], SiO_2_ [2], ZrO_2_ [3,4], and Al_2_O_3_ [3,5], but also Si_3_N_4_ [3], SiC [3], silver [3], hydroxyapatite [3], graphene [6], rubber [7], polyethylene [7,8], polyacrylonitrile [9,10], and glass fibres [8,11,12]. However, none of these compounds significantly increased strength or combined well enough with PMMA leading to a rather poor mechanical performance of removable dentures.

Cellulose is a natural polymer, which is non-toxic for humans. It is, therefore, possible to be used in combination with dental acrylic resin [13]. Moreover, cellulose is relatively cheap when compared to other fillers. The composites of PMMA filled with cellulose showed considerable improvement of the mechanical properties: elongation at break was changed from 1.6% to almost 3.5%, breaking strength increased by around 0.5 MPa, reaching value 3.75 MPa, while those parameters were improved with the addition of 1% of modified cellulose only [14].

Other studies demonstrated that impact strength was improved from 2.7% to 22.9% [15,16]. The tensile strength increased by 30% with a 150% increase in Young’s modulus when 10 wt% of the modified cellulose was added [17,18]. 

The main problem of cellulose application as the reinforcing filler is the lack of compatibility between the filler and the matrix. The chemical nature of these chemical species excludes their possible interactions. Cellulose is very hydrophilic. Water molecules can easily attach to the cellulose hydroxyl groups, which are located on the carbons in the glucose unit of cellulose. Due to its nature [19], cellulose tends to agglomerate when it is introduced into hydrophobic poly(methyl methacrylate), hence its uniform dispersion is impossible. Therefore, it is necessary to modify its surface in such a way that it demonstrates the more compatible character with the polymer used as a matrix for the denture plate. 

There are many different approaches to how the modifications could have proceeded. For example, cellulose nanocrystals were modified by the polymerisation of styrene via surface-initiated atom transfer radical polymerisation (SI-ATRP) [14]. Alternatively, radical mediated oxidation with 2,2,6,6-tetramethylpiperidin-1-oxyl (TEMPO) was used [16]. The same reagent and also the same method were used to modify cellulose nanofibres (CNFs) [17]. In another approach, the surface of CNFs was treated with methyl methacrylate (MMA), followed by in situ suspension polymerisation [18].

Silanes are very common coupling agents used for inorganic surface modifications. Several examples were demonstrated to be efficient for cellulose, such as trimethoxysilanes containing vinyl- [20,21], 3-aminopropyl- (APTMS) [22], methyl- (MTMS), propyl- (PTMS), hexadecyl groups (HDTMS) [23], or triethoxysilanes containing 3-aminopropyl- (APTES) [24,25] or octadecyl- substituents [26]. In most cases sol–gel chemistry was used for the modifications with silanes, although the melt-extrusion technique was also reported [27]. 

To sum up, based on the literature reports, it was assumed the modified cellulose could be used as an active filler of PMMA to be used for removable dentures. Herein, in this study, a cheap and straightforward way of cellulose modification was applied using octyltriethoxysilane (OTES) and (3-methacryloxypropyl)methyldimethoxysilane (MPMS). Obtained cellulose was further implemented into the dental acrylic resin. Due to the hydrophobic character of cellulose, better dispersion of modified cellulose in PMMA, as well as its contribution to the polymerisation of MMA through (3-methacryloxypropyl) groups was obtained. As a result, enhanced mechanical properties of dental acrylic resin were achieved. The application of cellulose into the dental resins has not been reported so far. Furthermore, the procedure of cellulose modification is straightforward, therefore, the potential of the practical implementation of this approach is very high. 

## 2. Materials and Methods

### 2.1. Materials

Arbocel^®^ UFC100 Ultrafine Cellulose for Paper and Board Coating cellulose microfibres (CMFs) in microfibre form with average length 8–30 μm were purchased from the J. Rettenmaier USA LP Company, Schoolcraft, MI, USA and used as received. Silanes, OTES, 97%, obtained from the Sigma-Aldrich, Saint Louis, MO, USA, MPMS, 92%, from the ABCR GmbH, Karlsruhe, Germany were used without any purification. Monomers: MMA, 99%, Alfa Aesar Company, Ward Hill, MA, USA, and ethylene glycol dimethacrylate (EGDMA), 98%, Sigma-Aldrich, were used as received. Heat-cured powder of acrylic resin Vertex^®^ Rapid Simplified was purchased from the Vertex Dental Company, Zeist, The Netherlands. All the solvents, acetone (Eurochem BGD Company, Tarnów, Poland), methanol (Chempur, Karlsruhe, Germany), and hexane (Chempur, Karlsruhe, Germany) were pure grade and used as received. 

### 2.2. Modification of Cellulose

**Figure 1:**
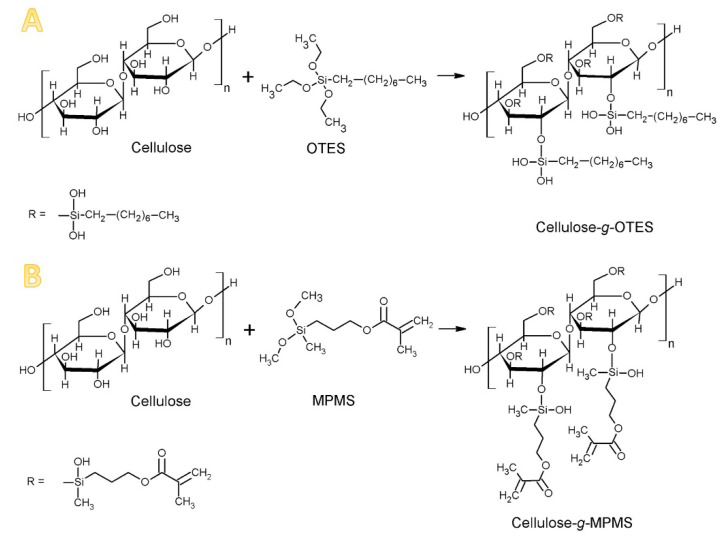
Schematic presentation of the modification of cellulose unit by (**A**) octyltriethoxysilane (OTES) and (**B**) (3-methacryloxypropyl)methyldimethoxysilane (MPMS).

Two types of silanes were used: OTES and MPMS. The protocol was the same in each case; 15 mmol of silane was used per 20 g of cellulose. Before the silanization process, cellulose was dried at a temperature of 100 ℃ for 60 min. The dried cellulose (20 g) was dispersed in 200 mL of acetone using ultrasounds. Subsequently, 4.7 mL of OTES or 3.5 mL MPMS were added to the CMFs dispersion. The dispersion was left while mixing for 30 min and next moved to the rotary evaporator. The mixture was kept there at 40 ℃ for 120 min with a rotation speed of 60 r/min. After that, the solvent was evaporated until the dry powder was obtained. The modified CMFs were dried in a vacuum dryer at 100 ℃, 17,000 Pa for 240 min. Schematic presentations of both chemical reactions are shown in Figure 1.

### 2.3. Preparation of Dental Composites

**Figure 2:**
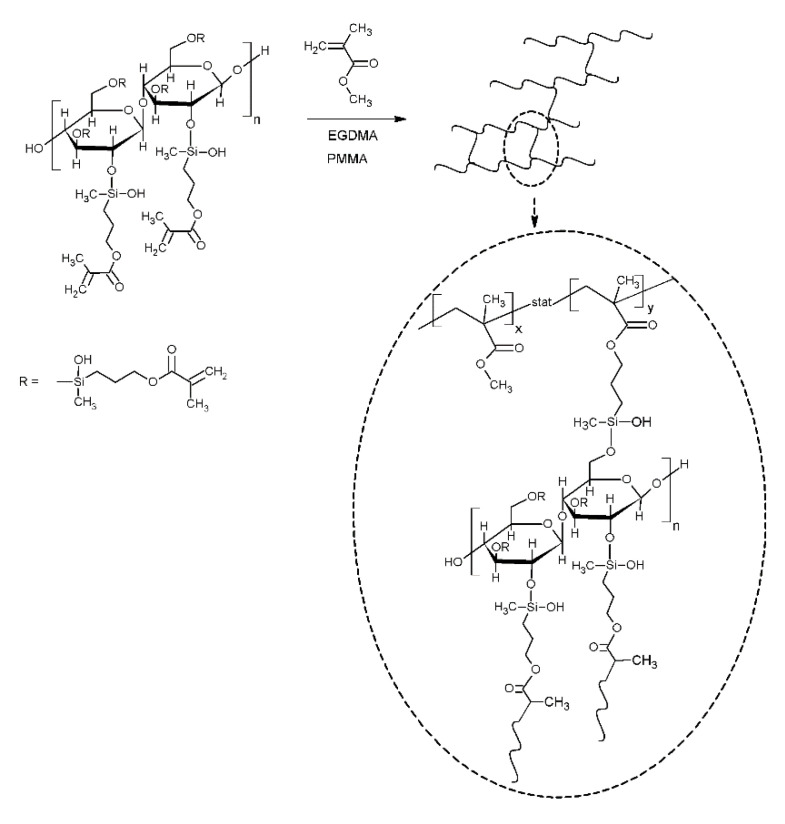
Scheme demonstrating the incorporation of modified cellulose into poly(methyl methacrylate) (PMMA) matrix.

Dental composites were prepared according to the recommendation provided by the supplier; 1 mL of liquid component (MMA monomer with crosslinker EGDMA) should be used per 2.3 g of powder (PMMA, containing polymerisation initiator). Herein, the powder was mixed with modified cellulose (1 g cellulose per 34.5 g powder) before the polymerisation (Figure 2), while the liquid was the mixture of MMA and EGDMA (according to the supplier specification: vol MMA/vol EGDMA = 95/5; vol/vol). Next, the liquid was poured into the powder; the content was mixed and left for 15 min for the pre-polymerisation to happen. The mass was then placed in the forms. The polymerisation proceeded at 100 ℃ for 20 min; after that, the specimens were left for air cooling to achieve ambient temperature.

### 2.4. Methods 

Fourier transform infrared spectroscopy (FTIR) analysis was carried out with a Nicolet 6700 FTIR spectrometer (Thermo Electron Company, Waltham, MA, USA). The spectra were recorded at a range of 4000–600 cm^−1^ with a resolution of 4 cm^−1^, using 32 scans. The weight gain and surface free energy (SFE) were measured with Force Tensiometer K100 (KRÜSS GmbH Company, Hamburg, Germany) according to the Washburn equation and Darcy’s law [28,29]. Weight gain was measured for packed cellulose powder in a glass tube being in contact with used liquid. Weight changes were calculated using heptane and methanol as solvents. The morphology evolution of composites was investigated using a JSM-6610LV Scanning Electron Microscope (SEM) (JEOL USA Company, Peabody, MA, USA). Differential scanning calorimetry (DSC) analysis of composites was performed in a temperature range of −50 ℃ to 200 ℃ with a heating rate of 10 ℃/min under inert atmosphere (nitrogen flow rate 10 mL/min) using TGA/DSC apparatus Mettler Toledo (Columbus, OH, USA). Thermogravimetric analysis (TGA) was prepared in a temperature range from 25 ℃ to 800 ℃ in an argon atmosphere, a flow rate of 60 mL/min, with a heating rate of 10 ℃/min, using TGA/DSC apparatus Mettler Toledo (USA). The swelling measurements were carried out using acetone as a solvent for 72 h, following the ASTM D6814-02 standard. A 3-point bending test was conducted with the used universal testing machine (from Zwick/Roell, Ulm, Germany) with the speed of test 1 mm/min. The specimens had the dimensions of 8 mm × 8 mm × 95 mm. 

## 3. Results

### 3.1. Fourier Transform Infrared Spectroscopy (FTIR) Analysis

**Figure 3:**
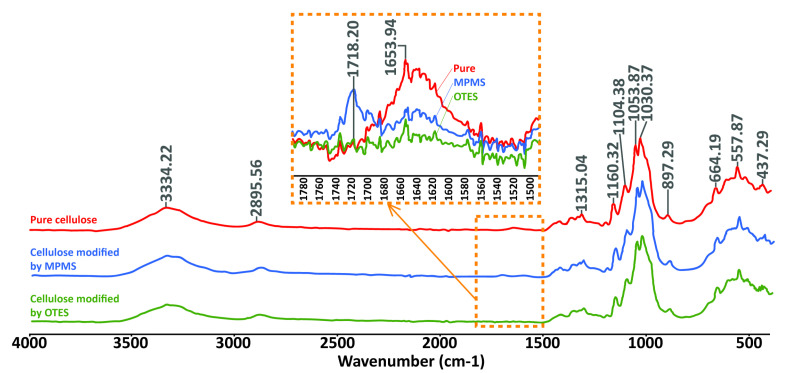
FTIR spectra of cellulose before and after the modification with silanes.

The FTIR spectra of the cellulose before and after modification with OTES and MPMS are given in Figure 3. 

The following bands were identified for the raw cellulose: ≈3300 cm^−1^, 2900 cm^−1^, 1600 cm^−1^, 1300 cm^−1^, 1100–1000 cm^−1^, 900 cm^−1^, and 700–400 cm^−1^. After modification, the shifts of some bands were observed. The most important feature was the appearance of a new band at 1750–1700 cm^−1^, indicating the successful modification of cellulose.

### 3.2. Sorption and Surface Free Energy (SFE)

**Figure 4:**
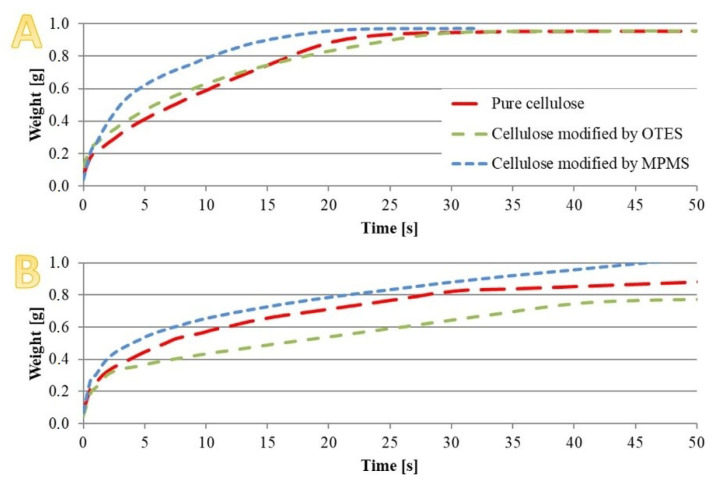
The normalised weight of cellulose wetted with (**A**) heptane and (**B**) methanol.

The changes in cellulose surface character resulted in the variation of cellulose wettability. Figure 4 presents the normalised weight of the investigated cellulose as a function of time for the samples wetted with methanol (Figure 4A) and heptane (Figure 4B), respectively. 

Surface free energy was also calculated based on the Owens, Wendt, Rabel and Kaelble (OWRK) method [30] but no significant differences for particular systems were observed. The calculated values for both unmodified and modified cellulose were similar: 20.6 mN/m for pure cellulose, 21.2 mN/m for cellulose modified by using OTES and 22.1 mN/m for cellulose modified with MPMS, respectively. 

### 3.3. Scanning Electron Microscope (SEM)

**Figure 5:**
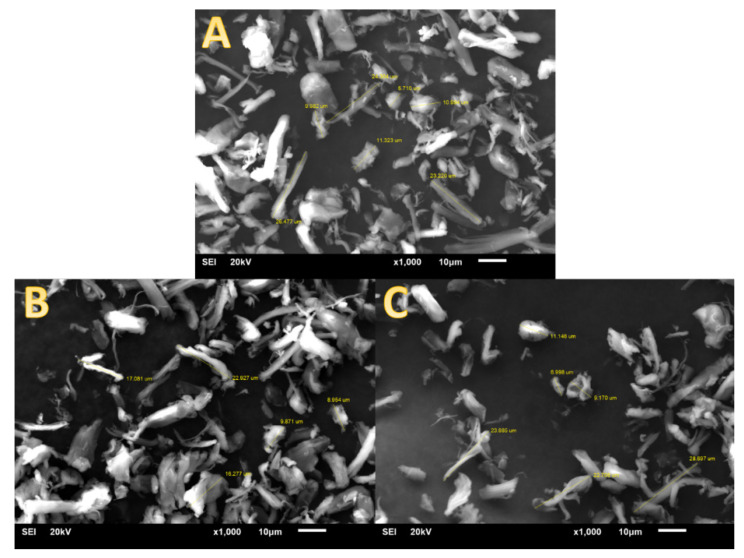
SEM images of (**A**) pure cellulose, cellulose modified with (**B**) OTES and (**C**) MPMS, at magnification 1000×.

The morphology of cellulose particles was verified by SEM. Figure 5A–C show micrographs of the pure CMFs, modified with OTES or MPMS, respectively. A detailed analysis of single particles allowed us to determine the length of the particles to be from around 8 μm to 30 μm in each case.

**Figure 6:**
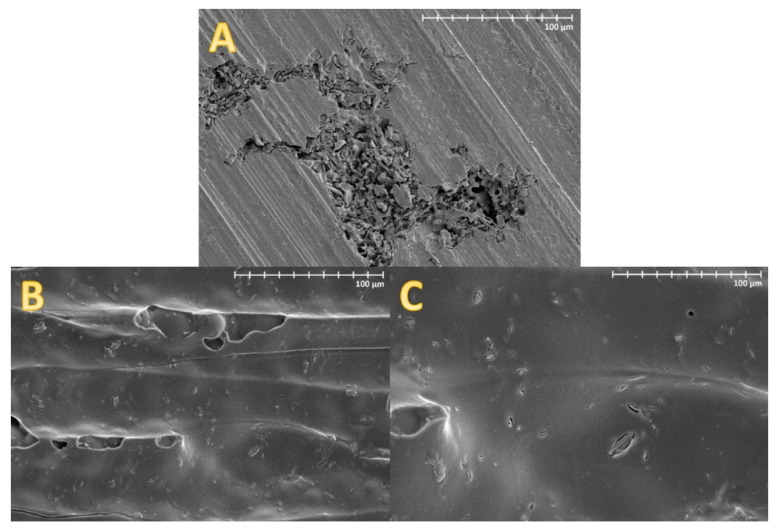
SEM images of acrylic resin with the addition of (**A**) unmodified cellulose, and cellulose modified with (**B**) OTES and (**C**) MPMS, at magnification 1000×.

Figure 6 shows the SEM images of prepared dental composites. Similar SEM images were observed in the case of both silanes.

### 3.4. Differential Scanning Calorimetry (DSC)

**Figure 7:**
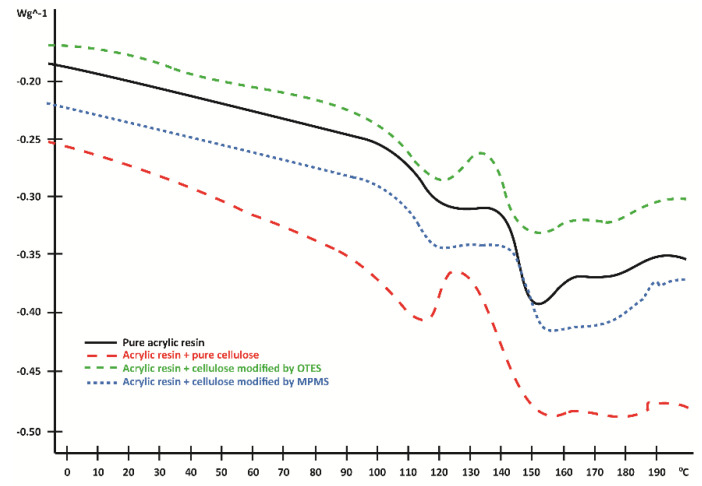
Differential scanning calorimetry (DSC) diagrams of investigated materials.

Figure 7 shows the results of the DSC analysis for investigated materials: pure acrylic resin, pure acrylic resin with the addition of raw cellulose, or modified one. All the investigated systems demonstrated the double phase transition. This effect results from the presence of two types of PMMA that differ by molecular parameters (molecular weight, dispersity, and cross-linking degree). One phase is the matrix, dental acrylic resin, which has already been defined independently. The other phase is formed within the reaction mixture based on the polymerisation conditions for monomer and crosslinker specified for the current studies. 

### 3.5. Swelling Measurement

**Figure 8:**
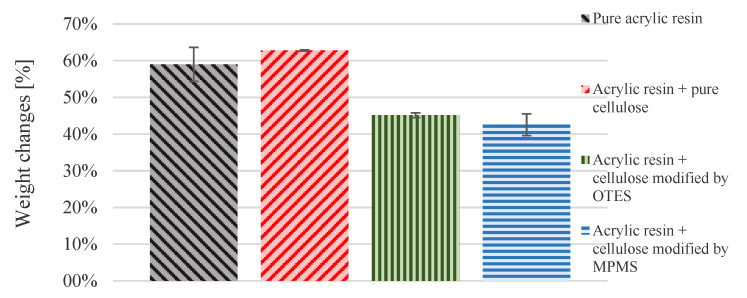
Weight changes (%) of analysed samples.

Figure 8 demonstrates the swelling results for all the investigated samples in acetone. Swelling affinity is reflected by the weight increase after the determined time (48 h). Acetone was selected as a good solvent for PMMA. Specimens of acrylic resin with pure cellulose showed the highest swelling tendency on the contrary to the sample containing cellulose modified with MPMS. In the last case, the smallest swelling was observed.

### 3.6. Thermogravimetric Analysis (TGA)

**Figure 9:**
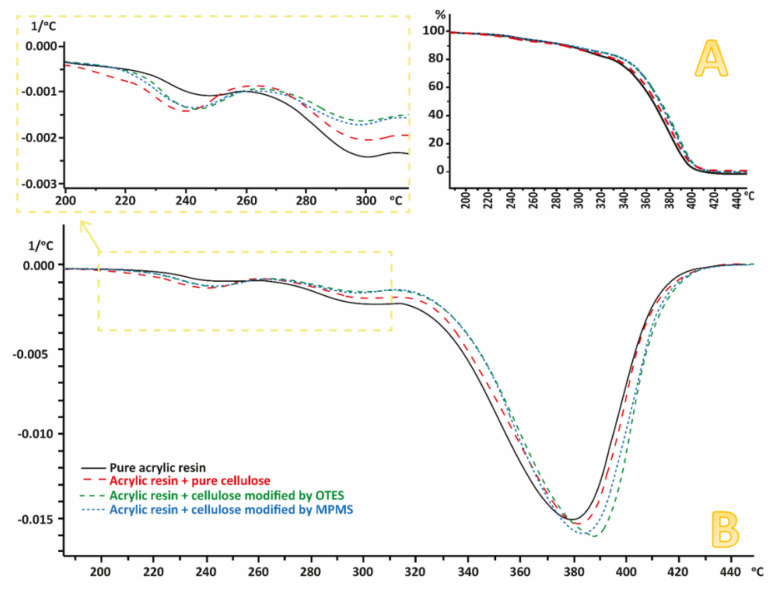
Results of TGA measurements: (**A**) the percentage of weight loss, (**B**) derivative of weight loss.

The results of the TGA analysis are shown in Figure 9. Although the content of the silane was relatively small, those compounds influenced the thermal stability of the prepared specimens. The decomposition temperature moved by a few degrees; the curves corresponding to the weight loss with temperature are clearly separated (Figure 9A). Furthermore, the temperature of the peak of derivatives moved from 395.95 ℃ to 399.76 ℃ and 402.37 ℃, respectively. The weight variations between 220 ℃ and 300 ℃ are connected with the depolymerisation of the PMMA matrix.

### 3.7. Three-Point Bending Test

**Figure 10:**
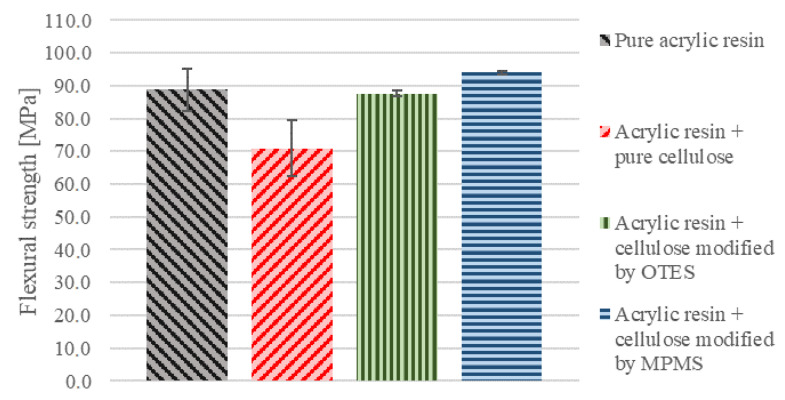
Flexural strength of analysed samples.

Results of the three-point bending test as seen in Figure 10 correspond to our earlier research [31]. The presence of raw cellulose did not improve the mechanical performance of the obtained composite; the flexural strength of the sample did not increase significantly. However, the application of silane MPMS for cellulose modification led to better performance of obtained composites. Flexural strength increased satisfactorily, which may be the result of better distribution of the particles within the polymer matrix.

## 4. Discussion

Cellulose microfibres, as a renewable material that can be used as reinforcing filler in composite materials, has been the object of growing interest for many years [13,14,15,16,17,18]. This is because in many applications, its bio-friendly character is one of its crucial features. However, the field of dental composites has not been explored that much from the point of view of cellulose applications. That is mostly because cellulose suffers from its poor dispersibility in the hydrophobic PMMA matrix, similar to many other examples [18,19,32,33]. Herein, the attention has been focused on the procedure of cellulose modification that is easy to be implemented for the preparation of the dental acrylic resins. Two types of silanes were used within current studies, OTES and MPMS. Although OTES is only responsible for the reduction of hydrophilic character of the cellulose, MPMS is a bifunctional molecule, which is immobilised on the cellulose microfibres through alcoxy groups, leaving the methacryloxy group available for radical polymerisation during dental acrylic resin formation. The concept of the cellulose modification with functionalised silanes was already reported [20,21,22,23,24,25,26] but herein, the procedure was simplified to the thermal treatment of the cellulose dispersion in acetone together with a particular silane. 

The effectiveness of the cellulose modification was verified by FTIR spectroscopy, Figure 3. The pure cellulose presents the characteristic peaks at 3600–2900 cm^−1^ that are assigned for stretching vibration of O–H and –C–H bonds in polysaccharides. A broad peak around 3300 cm^−1^ in Figure 3 of the FTIR measurement is not only because of the stretching vibration of hydroxyl groups but also because of the inter- and intramolecular hydrogen bonding vibrations in cellulose. Cellulose also demonstrates the characteristic peaks in the region 1600–900 cm^−1^. The peak around 1600 cm^−1^ refers to the water molecules absorbed on the cellulose. In the area of wavelength between 1500 and 1350 cm^−1^, CH_2_ and –CH groups can be detected. Peaks marked as 1315 cm^−1^ are –OH groups. The band around 1430–1420 cm^−1^ is assigned to the crystalline structure of cellulose; on the contrary, the band around 890 cm^−1^ corresponds to the amorphous phase of the cellulose. The peaks near 1100 cm^−1^, 1050 cm^−1^, and 1030 cm^−1^ visible for raw cellulose may overlap with unhydrolysed –Si–OCH_3_ and –Si–O–Si–, formed through condensation of silanol groups. The area from around 700 cm^−1^ corresponds mostly to the C–O–C, C–O, C=O or –CH and side group vibrations [34,35,36,37,38,39]. However, the most significant differences were observed in the area between 1750 and 1550 cm^−1^. In the case of cellulose modified with MPMS, a new peak appeared in the area between 1750–1700 cm^−1^. This is because of the carbonyl stretching absorption, which is one of the most typical bands used for the characterisation of carbonyl-containing compounds. The peak around 1650–1600 cm^−1^ [34,39] decreases its intensity in both modified cellulose and, in cellulose modified with OTES, it disappears completely. In this region, at ≈1600 cm^−1^ bands of C=C groups of acrylic species appear, similar to water, so it was not possible to detect them separately. Overall, the spectra identification confirms the successful cellulose modification by particular silanes.

The changes of microfibres’ surface character after silane treatment resulted in varied behaviour when in contact with liquids with different polarity, heptane and methanol in this case. Measurement of swelling was calculated according to the literature [28,29]. In the case of heptane, which is nonpolar, the most significant changes were observed in the case of cellulose modified with MPMS. Its full wettability was achieved within the shortest time; on the contrary, the pure cellulose required the longest time (Figure 4). Mass gain, with time, was always the lowest in the case of cellulose-containing tethered octyl groups, as shown in Figure 4B. It should be noted that not only the different polarity of MPMS and OTES but also varied surface coverage by those two silanes could lead to the different dynamic of the cellulose weight changes because methoxy or ethoxysilanes demonstrate diverse activity in the reaction of their immobilisation on cellulose.

Alcoxysilanes, due to their multifunctionality (di- or trialcoxy-) could also create a continuous silica layer around single cellulose fibres [40,41,42,43]. However, if the modification procedure is right, the formation of siloxane bonds may be suppressed and the immobilisation of a silane monolayer on the cellulose surface is predominantly observed [41,42,43]. Based on SEM observation from Figure 5, a multilayer of silica was not detected. Detailed analysis of single-particle morphology showed the dimensions of the microfibres are consistent with the information on the manufacturer’s leaflet [44]. Therefore, it can be concluded that the shape and size of the particles do not differ after the modification. 

The prepared dental resins were filled with the same amount of silane-modified cellulose microfibres to observe their reinforcing effect. The uniform distribution of the filler within the matrix, PMMA, is significant in such a case [31]. Figure 6 shows the SEM images of the dental composites containing cellulose. It is visible that the unmodified cellulose tends to agglomerate, resulting in poor mechanical properties of the final material as it was already demonstrated [19]. On the contrary, the surface modification allows uniform distribution of the microfibres in the matrix. A similar effect was previously reported in the literature [45]. 

The presence of additional functional groups such as 3-methacryloxypropyl provides the opportunity to combine the filler and the polymer matrix through the covalent bond, thus enhancing the compatibility of those two phases [20,21,22,25,26,46]. DSC measurements (Figure 7) showed that two phase transitions are present in all investigated dental composites. The first transition, which occurs at the lower temperature range, is associated with the already polymerised PMMA (onset 106.02 ℃, midpoint 114.54 ℃). The presence of CMFs caused the onset of *T_g_* temperature to move towards a lower temperature range (onset 97.51 ℃, midpoint 109.30 ℃). This could suggest that these particles acted as plasticisers for this phase [46]. This effect was decreased if silanes were tethered on cellulose leading to some interactions with the polymer matrix; hence, the onset was moved to 102.04 ℃ and 105.44 ℃ for OTES and MPMS, respectively. In the case of the second phase, similar trends were observed. The most significant shifts of the *T_g_* temperature were detected for the system when silane (MPMS) contributed to the polymerisation and cross-linking of the resin, 143.92 ℃. In the case of a sample containing OTES groups and pure cellulose, this was 135.16 ℃ and 128.81 ℃, respectively. However, especially for the second transition, it is expected that the differences reflect not only the influence of silanes but also the variation of cross-linking density generated by the presence of methacryloxypropyl groups. 

To quantify the changes in cross-linking density, the swelling measurements were recorded. The higher the cross-linking density was, the lower the weight increment was observed. Figure 8 shows the samples’ weight changes after swelling in acetone. This confirms that the methacryloxypropyl groups contributed to the radical polymerisation of MMA, also leading to the cross-linking of formed resin, Scheme 1. Specimens of acrylic resin with pure cellulose showed the highest swelling tendency, which means the cross-linking degree was the lowest in this sample. 

Although the content of the silane was relatively small, those compounds influenced the thermal stability of the prepared specimens. Similarly to the published results [46], the decomposition temperature of PMMA resin changed after the addition of cellulose. The curves corresponding to the weight loss of composites moved towards higher temperatures, Figure 9A. Furthermore, the maximum temperature of the peak of derivatives moved from 395.95 ℃ to 399.76 ℃ and 402.37 ℃, respectively, for composites containing cellulose modified with particular silanes. 

Based on the mechanical tests, it was concluded the presence of cellulose is not the exclusive requirement for the better performance of obtained dental resins. Only in the case of cellulose modified with MPMS, the improvement of flexural strength of dental acrylic resin was detected and the value changed from 70.9 MPa to 94.0 MPa (Figure 10). As already discussed, this value is influenced by the cross-linking degree [47]. Similar systems reported in the literature demonstrated flexular strength in the range of 2.50 GPa to even 3.5 GPa when fibre length was 3.0 mm [13]. However, as it was already pointed out, the flexural strength values obtained for varied specimen cannot be compared directly because this parameter depends on the specimen size, the specimen tensile strength, and material fracture energy. Small specimens may tend to withstand higher local stresses than constructive material. This is because damage requires the collection of enough energy to be released into the crack surface creation energy [48]. Nevertheless, based on the obtained results, it is expected that the optimisation of the cellulose modification process in the future can provide better effects.

## 5. Conclusions

Octyltriethoxysilane and (3-methacryloxypropyl)methyldimethoxysilane were effective modifying agents for cellulose. The cellulose character was changed into less hydrophilic, therefore, better distribution of cellulose particles within poly(methyl methacrylate) dental resin was achieved as proved by microscopic analysis. As a result, the obtained composites demonstrated improved mechanical properties and higher thermal stability. It is considered that (3-methacryloxypropyl)methyldimethoxysilane is a better modifying agent because it contributed successfully in both the polymerisation and cross-linking reactions; therefore, the compatibility of the cellulose and poly(methyl methacrylate) was enhanced. Further optimisation of silanes functionality and cellulose modification conditions are on the way.
